# Effects of *Staphylococcus aureus* on stem cells and potential targeted treatment of inflammatory disorders

**DOI:** 10.1186/s13287-024-03781-6

**Published:** 2024-06-27

**Authors:** Zi-xian Liu, Guan-qiao Liu, Ze-xin Lin, Ying-qi Chen, Peng Chen, Yan-jun Hu, Bin Yu, Nan Jiang

**Affiliations:** 1 Division of Orthopaedics & Traumatology, Department of Orthopaedics, Southern Medical University Nanfang Hospital, Guangzhou, 510515 China; 2 Guangdong Provincial Key Laboratory of Bone and Cartilage Regenerative Medicine, Southern Medical University Nanfang Hospital, Guangzhou, 510515 China; 3 Department of Orthopedics, Lanzhou University Second Hospital, Lanzhou, 730000 China

**Keywords:** Staphylococcus aureus, Mesenchymal stem cells, Adipose stem cells, Inflammatory disorders

## Abstract

Due to the advanced studies on stem cells in developmental biology, the roles of stem cells in the body and their phenotypes in related diseases have not been covered clearly. Meanwhile, with the intensive research on the mechanisms of stem cells in regulating various diseases, stem cell therapy is increasingly being attention because of its effectiveness and safety. As one of the most widely used stem cell in stem cell therapies, hematopoietic stem cell transplantation shows huge advantage in treatment of leukemia and other blood-malignant diseases. Besides, due to the effect of anti-inflammatory and immunomodulatory, mesenchymal stem cells could be a potential therapeutic strategy for variety infectious diseases. In this review, we summarized the effects of *Staphylococcus aureus* (*S. aureus*) and its components on different types of adult stem cells and their downstream signaling pathways. Also, we reviewed the roles of different kinds of stem cells in various disease models caused by *S. aureus*, providing new insights for applying stem cell therapy to treat infectious diseases.

## Introduction

As one of the most frequently detected bacteria in human infectious diseases [1], *S. aureus* is closely related to the occurrence of lung infection, bacteremia, infective endocarditis, osteomyelitis, and many other inflammatory disorders [2]. The preferred treatment for *S. aureus*-related infection is β-lactam antibiotics [3]. However, antibiotic resistance has developed rapidly in recent years. It has been reported that the death per year caused by antibiotic resistance has exceeded 10 million and will exceed that caused by cancer by 2050 [4]. Based on this emergency issue, exploring new strategies against such antibiotic resistance is crucial. Recently, many studies have reported that stem cells have powerful immune regulatory functions, playing an essential role in treating various infectious diseases [5].

Stem cells are a group of cells with self-renewal and self-differentiation functions [6], which are classified into four categories according to their sources: embryonic stem cells (ESCs), fetal and adult stem cells, and induced pluripotent stem cells (iPSCs) [7]. Since adult stem cells do not cause rejection or ethical controversy, relevant studies on them have been applied in the models of various infectious diseases [8]. Although many studies have confirmed that stem cells play positive roles in infectious diseases, they can also be influenced by the infectious environment. Understanding the mechanisms of how *S. aureus* and its components affect the functions of stem cells may help us exert their anti-infectious function more effectively and play more excellent value in the management of infectious diseases.

## Effects of *S. Aureus* on stem cells

### Mesenchymal stem cells (MSCs)

**Fig. 1:**
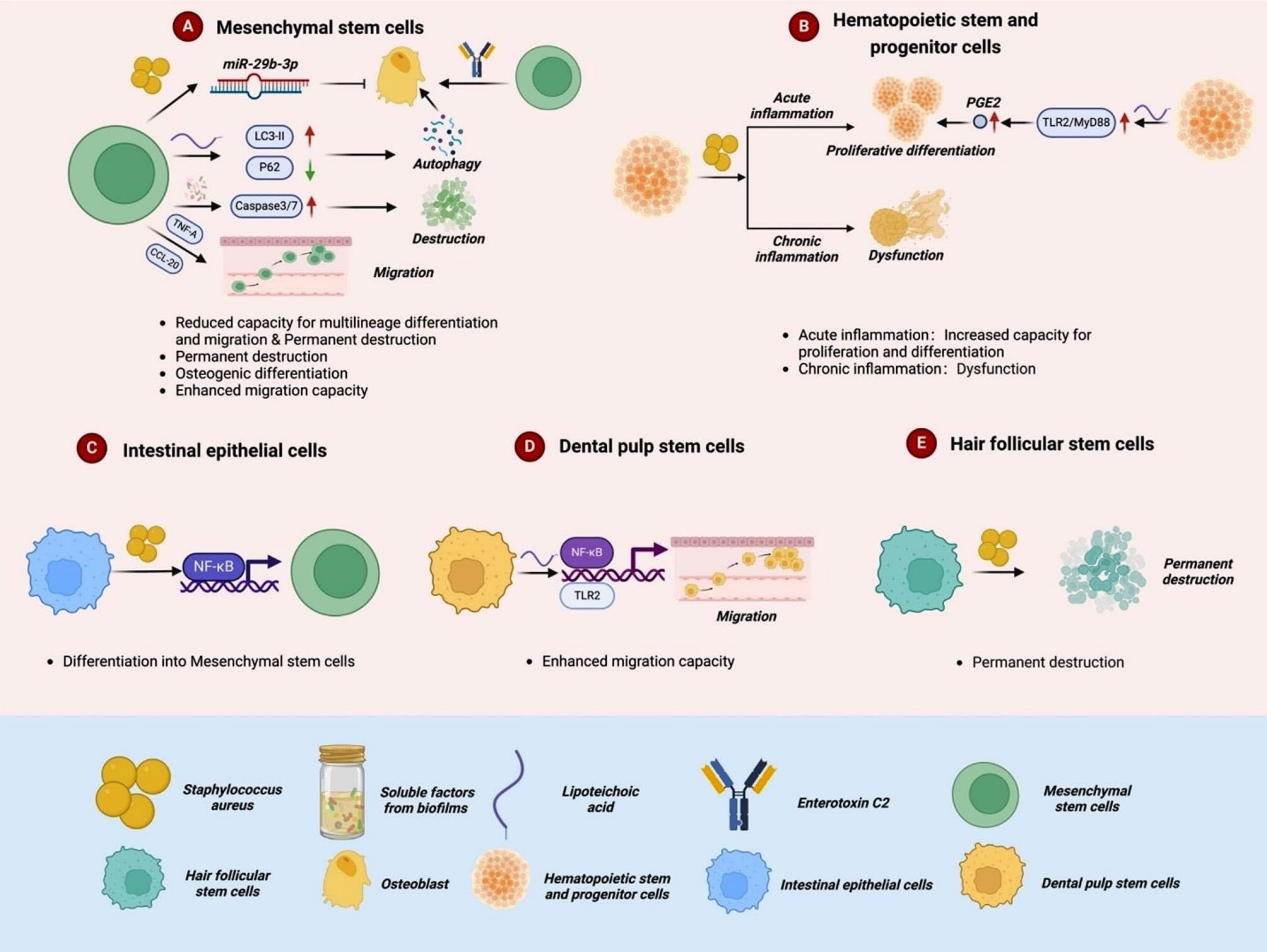
The mechanism of how *S. aureus* interacts with stem cells

As a common pluripotent stem cell, MSCs have recently received extensive attention in regenerative medicine, among which bone marrow-derived mesenchymal stem cells (BMSCs) are the most widely investigated [9]. In addition to bone marrow, MSCs can be isolated from different mature tissues, such as skeletal muscle, adipocytes, umbilical cord, amniotic fluid, peripheral blood, intima synovium, dental pulp, lung, and liver [10, 11]. Such MSCs can differentiate into bone, chondrocytes, fat, muscle, neurons, islet cells, and liver cells under specific conditions [10–12]. Recent studies have found that *S. aureus* can affect migration and recruitment of lineage differentiation and activity of MSCs (Fig. 1; Table 1).

#### Migration

As an essential therapeutic tool in regenerative medicine, MSCs have been proven to enhance proliferation during tissue injury, inflammation, and tumorigenesis. Then, they differentiate into different types of cells under different microenvironment stimulations and participate in tissue repair [13, 14]. Recent studies in vitro have indicated that the number of MSCs at the site of infection caused by *S. aureus* significantly increased, possibly due to the local inflammatory specificity that promotes the migration and implantation of MSCs [15, 16]. Yang et al. [17] indicated that the intestinal epithelial cells alone did not affect the migration of cord blood-derived MSCs. In contrast, the intestinal epithelial cells infected with *S. aureus* significantly enhanced the above biological process. It suggests that *S. aureus* may regulate the migration of cord blood-derived MSCs by influencing the secretion of migration-related chemokines. Further analysis revealed that *S. aureus* enhanced the expressions of tumor necrosis factor α (TNF-α) and C-C motif chemokine ligand 20 (CCL20) by activating the NF-κB signaling pathway in the intestinal epithelial cells. However, Ward et al. [18] found that the conditioned medium to produce *S. aureus* biofilm resulted in an increased caspase 3/7 activity in primary human bone marrow stromal cells (hBMSCs), leading to apoptosis and loss of viability. They also observed that the migration ability of cells exposed to soluble factors in the biofilm was significantly lower than that in the control group. However, the expressions of wound-healing promoting cytokines, such as stromal derived factor 1 (SDF-1) and vascular endothelial growth factor (VEGF), and antimicrobial peptides LL-37, were significantly increased. It demonstrates that the migration ability of MSCs differed among different stages of infection caused by *S. aureus*. During the acute phase, the inflammatory factors and chemokines produced by *S. aureus* may promote the migration of MSCs. However, after the establishment of bacterial biofilm, it can inhibit MSCs migration and reduce MSCs activity through the up-expression of wound-healing associated factors. These results imply that the formation of *S. aureus* biofilm impairs cells’ ability to recruit MSCs after bacterial stimulation, which provides new targets and therapeutic strategies for clinical diagnosis and treatment.

#### Osteogenic and adipogenic differentiations

Fractures, one of the most common orthopedic diseases, usually take over three months to heal. However, about 10-20% of patients still suffer from delayed union or non-union. One of the critical factors influencing bone healing is bone remodeling [19, 20], a dynamic process between osteoblast (bone formation) and osteoclast (bone resorption) [21]. During this process, MSCs play an essential role owing to the differentiation abilities of osteogenesis, chondrogenesis, and adipogenesis [22]. In recent years, many studies in vitro have indicated that bacteria and their components significantly impact the differentiation of MCSs in osteogenesis and adipogenesis, among which *S. aureus* is one of the most frequently analyzed [18, 23–26]. Ding et al. [23] found that, after infecting hBMSCs with different concentrations of *S. aureus* (0, 0.5, 1, 10, and 50 µg/ mL), the expressions of osteogenesis genes were inhibited after 14-days osteogenesis differentiation in a dose-dependent manner. They also found that the expression of miR-29b-3p in hBMSCs significantly increased. Moreover, when miR-29b-3p was inhibited, expressions of the osteogenesis genes in hBMSCs were noted to grow considerably, indicating that miR-29b-3p might be a negative regulator against hBMSCs osteogenic differentiation during infection [23]. As the rapid growth of bacteria in cell media leads to rapid nutrient depletion and acidification, Tomas et al. [24] used heat-inactivated *S. aureus* (HKSA) instead of alive *S. aureus* to conduct experiments. They observed that human adipose-tissue-derived mesenchymal stem cells (adMSCs) disposed of with HKSA significantly reduced the activity of alkaline phosphatase (ALP) as well as decreased adipogenic differentiation activity. Similarly, the soluble factors of biofilm produced by *S. aureus* in chronic infection played the same role. A previous study also showed that hBMSCs exposed to the conditioned medium of *S. aureus* biofilm significantly reduced the intracellular calcium deposits and oil droplets at 7, 14, and 21 days after osteogenic differentiation, with decreased expressions of osteogenesis and adipogenesis markers [18]. These results indicate that *S. aureus* and its biofilms can affect MSCs functions by inhibiting osteogenic and adipogenic differentiation abilities.

However, as virulence factors of *S. aureus*, α-hemolysin, lipoteichoic acid (LTA), and Staphylococcal enterotoxin C2 (Sect. 2) may have opposite functions on MSCs. Our previous study in vitro has shown that administrated mice with 40 µg/ml α-hemolysin significantly induced bone destruction directly by suppressing osteogenesis by stimulating the expression of caveolin-1 and activated lipid rafts accumulation in BMSCs [27]. However, LTA, as one of the primary components of the cell wall of *S. aureus*, was not found to affect the proliferation and differentiation of adMSCs in vitro [24]. Liu et al. [25] found that 10 µg/mL LTA could enhance the autophagy activity of MSCs in mice, manifested as increased expression of LC3-II protein and decreased expression of P62, thus promoting the osteogenic differentiation of MSCs. And osteogenic differentiation genes, such as ALP, collagen type I (ColI), and runt-related transcription factor-2 (Runx2), were upregulated. The difference in conclusions between the two studies may be due to differences in LTA concentrations. Besides, Wu et al. found that Sect. 2 also had the same bone-promoting effect on MSCs. The results showed that Sect. 2 had no significant impact on the proliferation of rat BMSCs at different concentrations from 1pg/ml to 500pg/ml. However, the formation of calcium nodules in BMSCs was significantly increased, and the expressions of osteogenic markers such as ALP, Runx2, OCN, and OPN were upregulated [26]. These results indicate *that S. aureus* and its related components have inconsistent effects on the osteogenic and adipogenic differentiation of MSCs, suggesting that the inhibition of MSCs by *S. aureus* is not caused by virulence factors alone. The changes in bone mass caused by these virulence factors may provide new targets and strategies for osteoporosis prevention and osteogenesis promotion in clinical.

### Hematopoietic stem cells and progenitor cells (HSPCs)

In addition to maintaining hemostasis, such as oxygen and nutrient transport, and innate and adaptive immune responses, the cells in the blood generated by hematopoietic stem cells (HSCs) also can promote tissue regeneration and repair. HSCs can self-renewal and have a variety of differentiation potentials. Through asymmetric division, they can generate daughter cells that maintain the HSCs potential and a hematopoietic progenitor cell (HPCs). Although HPCs lose their self-renewal ability, they can further differentiate into various mature blood cells, such as red blood cells, macrophages, neutrophils, and T and B lymphocytes (T and B cells). Therefore, HSPCs are generally regarded as the cornerstone of the biological hematopoietic and immune systems [28–30].

As an essential part of the immune system, HSPCs can respond to the inflammatory environment in various ways. Recently studies have indicated that HSPCs are the first responder during infection, and various inflammatory factors released, such as TNF and interleukin-1 (IL-1), can affect the function of HSPCs [31, 32]. *S. aureus* is one of the main bacteria which cause human skin and soft tissue infection [33, 34]. According to the severity and duration of infection, *S. aureus* showed two different effects on HSPCs ultimately. When the infection is mild or at the early stage, it could induce various acute inflammatory changes in the microenvironment of the infection site, resulting in the rapid recruitment of HSPCs in the peripheral blood circulation to the infection situation, and enhancing the survival and proliferation and differentiation ability of local HSPCs, thus generating many immune cells (such as polymorphonuclear neutrophils, PMNs) to eliminate the inflammation caused by infection. However, when the infection is severe or enters a chronic stage, HSPCs would cause chronic inflammatory persistence and destroy the ecological niche, thus leading to the failure and dysfunction of HSPCs [31, 35–39].

In addition, *S. aureus* can also affect the function of HSPCs by directly targeting it (Fig. 1; Table 1). Many previous studies in vivo have shown that the lipopeptides and LTA of *S. aureus* could directly interact with toll-like receptor 2 (TLR2) on HSPCs, thus activating the TLR2/ myeloid differentiation factor 88 (MyD88) downstream signaling pathway, leading to the enhancement of the proliferation and myeloid differentiation of HSPCs and increased the number of PMNs at the infection situation [40, 41]. By constructing TLR2 or MyD88 deficient transgenic mice, Granick et al. [42] also found little difference in the number of HSPCs in TLR2 or MyD88 deficient mice and WT mice. However, the number of HSPCs and its daughter cells (such as promyelocytes and PMNs) at the wound site in TLR2 or MyD88 defective mice infected with *S. aureus* showed an apparent decreasing trend, indicating that *S. aureus* does affect the functions of HSPCs by directly activating TLR2/MyD88 signaling pathway. By injecting isolated HSPCs from WT mice into the *S. aureus-infected* WT mice and TLR2 or MyD88 deficient mice, they found that HSPCs had similar proliferation and differentiation abilities in different wound environments. In addition, HSPCs from WT, MyD88, or TLR2 deficient mice pretreated with prostaglandin E2 (PGE2) were injected into wounds of *S. aureus*-infected WT mice and results showed TLR2-dependent PGE2 production could regulate the proliferation and differentiation of HSPCs in mice. Their study indicated that *S. aureus* could induce the production of PGE2 by directly activating the TLR2/MyD88 signaling pathway of HSPCs, which in turn target on HSPCs themselves through PGE2, thus increasing the survival, proliferation, and differentiation ability of HPSCs. This result improved the internal molecular mechanism of *S. aureus*, stimulating the proliferation and differentiation of HSPCs. In addition, Maneu et al. [43] co-cultured purified mouse bone marrow HPCs with inactivated *S. aureus* and found that it could directly induce the differentiation of HPSCs into the myeloid system. These results highlight the importance of HSPCs in anti-infection in *S. aureus* infection. Modulating the TLR2 signaling pathway or focusing on the severity or stage of the infection may provide a new strategy by intervening with the function of HSPCs in clinical infection.

**Table 1:** The role of *S. aureus* on mesenchymal, hematopoietic, dental pulp, and hair follicular stem cells

Cell type	Functional components	Mode of action	Outcomes
Mesenchymal stem cells(MSCs)	Unknown	Stimulated intestinal epithelial cells to secrete TNF-α and CCL20	Increased migration capacity of MSCs [17]
		Increased the expression of miR-29b-3p in hBMSCs	Inhibited osteogenic differentiation of hBMSCs [23]
	Soluble factors from biofilms	Increased the activity of caspase 3/7 of hBMSCs, which leads to cell apoptosis	Decreased migration capacity of MSCs [18]
		Unknown	Decreased multilineage differentiation capacity of MSCs [18]
	Lipoteichoic acid	Increased autophagy of MSCs	Promotion of the osteogenic differentiation of MSCs [25, 26]
	Staphylococcal enterotoxin C2	Unknown	
Hematopoietic stem and progenitor cells(HSPCs)	Unknown	Promotion of the formation of a systemic or local acute inflammatory microenvironment	Promotion of the survival, proliferation and differentiation ability of HSPCs and increased their migration ability [31, 32]
		Maintaining chronic systemic or local inflammatory states and disrupting the niche of HSPCs	Lead to HSPCs failure and dysfunction [31, 35, 37, 38, 128, 129]
	Lipopeptide, Lipoteichoic acid, etc.	Activation of the TLR2/MyD88 signaling pathway of HSPCs and promoted the secretion of PGE2	Promotion of the survival, proliferation and differentiation ability of HSPCs [40–42]
Dental pulp stem cells(DPSCs)	Lipoteichoic acid	Activation of TLR2/NF-κB signaling pathway in DPSCs	Promotion of the proliferation and migration ability of DPSCs [44]
Hair follicular stem cells	Unknown	Unknown	lead to permanent destruction of hair follicular stem cell [45, 46]

hBMSCs: Human bone marrow-derived mesenchymal stem cells; TNF-α: Tumor necrosis factor-α; CCL20: Chemokine (C-C motif) ligand 20; PGE2: Prostaglandin E2

### Dental pulp stem cells(DPSCs) and hair follicle stem cells

In addition to MSCs and HSPCs, *S. aureus* and its virulence factors can affect other stem cells in vitro differently (Fig. 1; Table 1). Shayegan et al. [44] analyzed the interaction between adult DPSCs and different concentrations of LTA. They found that LTA activated NF-κB signaling pathway through TLR2 in DPSCs, leading to the enhancement of proliferation and migration of DPSCs. In addition, as one of the most common pathogenic microorganisms in the human body, *S. aureus* can not only cause pulpitis, osteomyelitis, food poisoning, and other common inflammatory diseases but also be the leading cause of some inflammatory diseases, such as nail-free folliculitis (FD). As a rare scalp inflammatory disease, the pathogenesis of FD has been confirmed to be closely related to *S. aureus* infection and autoimmune dysfunction. Also, FD treatment primarily relies on antibiotics and immunomodulators. Studies have shown that FD can permanently destroy hair follicle stem cells, which indicates that *S. aureus* may adversely affect the function of hair follicle stem cells in the human body [45, 46].

## Effects of stem cells on *S. Aureus*

### MSCs

**Fig. 2:**
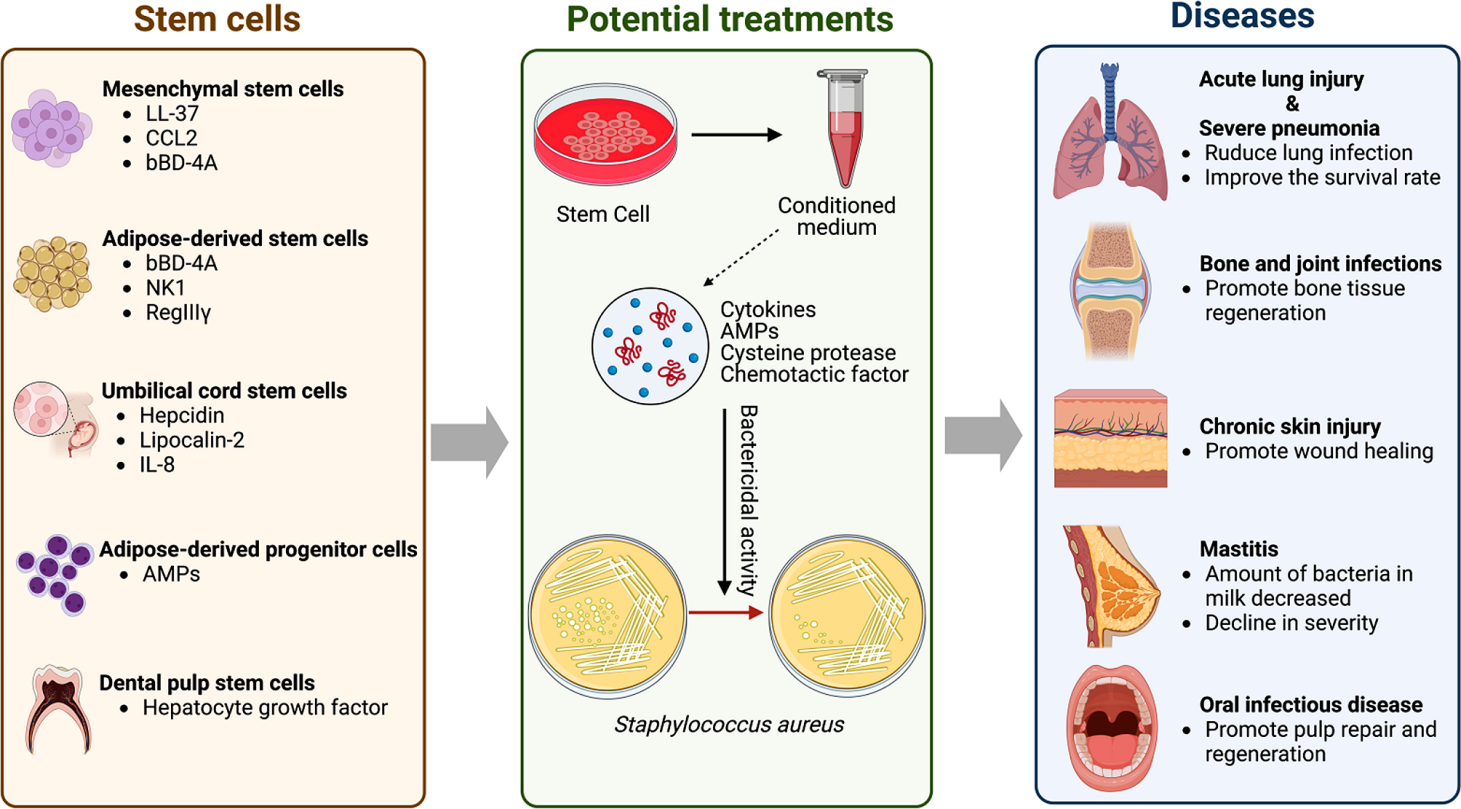
The role of stem cells in alleviating infectious and inflammatory disease

*S. aureus* infection is the primary microorganism that causes infections in the skin, soft tissue, respiratory system, bone and joint, and vascular systems [1]. Due to antibiotics abuse and the developed ability of antibiotic resistance, the prevalence of methicillin-resistant *S. aureus* (MRSA), the mortality rate of sepsis, and other diseases have been increasing in recent years [4]. Given this public health problem, it is crucial to develop new treatment strategies. As the most widely distributed adult stem cells in the human body, MSCs have been proven to help the host resist bacteria and reduce tissue damage and inflammation [5]. In this part, we summarize the role of mesenchymal stem cells in various diseases caused by *S. aureus* and their potential therapeutic targets (Fig. 2; Table 2), providing new possibilities for cell therapy in treating infections and inflammatory diseases.

#### Pneumonia in cystic fibrosis (CF)

CF is a fatal genetic disease that is caused by the mutation of the cystic fibrosis transmembrane conduction regulator (CFTR) gene, resulting in the defective activity of the chloride channel and increased viscosity of mucus, leading to severe lung infection and inflammation [47–49]. In recent years, with the development of CFTR enhancers and other drugs, new progress has been made in treating CF. However, these new drugs still find it challenging to improve the pulmonary bronchiectasis and inflammation caused by bacterial colonization, which leads to severe pulmonary edema and even death [50]. The latest research results in vivo found that treating human mesenchymal stem cells (hMSCs) significantly alleviates the CF lung infection caused by *S. aureus* [51]. Further studies in vitro showed that the bioactive molecules in the supernatant of hMSCs reduced the overall bacterial load, and the antimicrobial peptides (e.g., LL-37) produced by hMSCs enhanced the sensitivity of bacteria to antibiotics, thus improving the ability of antibiotics to kill bacteria. In addition, they found that hMSCs with impaired CFTR function may produce fewer LL-37 [51], suggesting that mutation of the CFTR gene in patients’ MSCs may also be one reason why *S. aureus*-associated pneumonia was persistent. In conclusion, exogenous hMSCs may provide us with a unique strategy for the treatment of CF patients.

#### Chronic skin trauma

Skin is the largest organ in the human body, regulating body fluid and balancing body temperature and antibacterial infection. It is the body’s first defense to resist harmful external stimuli. Therefore, the integrity of the skin plays a crucial role in the homeostasis of the internal environment [52]. The incidence of chronic skin trauma has been increasing recently. Delayed healing or even non-healing of chronic wounds leads to long-term skin defects, which further affect the functions of other tissues and organs in the human body and heavily burden public medical resources. A common feature of chronic skin wounds is that they are colonized by pathogenic bacteria such as *S. aureus*. The wounds infected by *S. aureus* generally show expansion and delayed healing [53]. At the same time, MSCs can be anti-inflammatory and bactericidal and promote tissue repair [54–56]. Therefore, the potential role of MSCs in chronic skin trauma attracts more and more attention.

Horses and humans all suffer chronic wounds due to *S. aureus* infection. The pathogenesis mechanism of delayed or non-healed is similar so that horses can be used as a physiologically related model of human wound healing [57, 58]. Harman et al. [59] used an equine MSC suspension and supernatant from healthy horses to culture *S. aureus*. They evaluated the growth of *S. aureus* and the damage to the bacterial membrane. The results showed that both the suspension and supernatant could effectively inhibit the growth of *S. aureus* and depolarize the bacterial membrane, leading to *S. aureus* biofilm destruction. At the same time, they also identified four specific antimicrobial peptides (AMP [60], a class of alkaline polypeptides with extensive antibacterial effects produced in multicellular organisms) secreted by equine MSCs, demonstrating that equine MSCs participated in antibacterial effects through the secretion of AMP. Subsequently, Marx et al. [61, 62] also used equine MSCs to study the therapeutic effect of MSCs on various chronic wound pathogens with biofilm-forming ability. They found that the MSCs could effectively inhibit the *S. aureus* biofilm formation in vitro by secreting cysteine protease. In addition, MSCs could also stimulate keratinocytes through the secretion of CCL2 to increase their AMP secretion capacity and antibacterial function, thus indirectly affecting the formation of *S. aureus* biofilm.

In addition to the horse model, mice and dogs commonly use physiologically related models for human wound healing. Johnson et al. [63] observed the effect of intravenous MSCs combined with antibiotics in treating chronic *S. aureus* wound infection in mouse and dog models and found that MSCs could effectively limit the severity of *S. aureus* infection in vivo and significantly enhance the efficiency of the antibiotics. Chow et al. [64] also studied the MSCs activity in the mouse biofilm infection model. They found that various cytokines secreted by MSCs could directly or indirectly target pathogenic microorganisms such as *S. aureus*, effectively inhibiting the microorganisms’ colonization in chronic wounds and thus improving wound healing.

Although MSCs have not yet been applied in clinical practice in the treatment of chronic wounds and the role of MSCs in delayed or non-healed human wounds caused by *S. aureus* infection is not clear, the antibacterial effect of MSCs in biological models such as horses, mice, and dogs provide a rich theoretical basis for the future application of MSCs in the treatment of chronic wounds.

### Adipose stem cells and progenitor cells

#### Adipose tissue-derived stem cells (ASCs)

ASCs, also known as adMSCs, are a particular type of MSCs that can be directly extracted from adipose tissue obtained through lipoplasty or liposuction without further expansion in a culture medium [65]. As one of the primary sources of mature adipocytes in adipose tissue, ASCs can self-renewal and have various differentiation potentials. Recently studies have shown that ASCs have the function of anti-inflammatory, cell microenvironment protection, and tissue regeneration [66, 67]. Therefore, its potential target role in treating various inflammatory diseases is gradually attracting research attention.

##### Chronic skin wounds

A recent case report on chronic venous leg ulcers showed that local autologous ASCs-enriched, high-density lipoaspirate (HDL), and timolol could promote the healing of ulcerative wounds [68]. In addition, Moradi et al. [69] also found that ASCs usage could significantly boost the healing of MRSA-infected wounds in type 2 diabetic rats model. Their findings suggest that ASCs may have a therapeutic effect on the delayed recovery of chronic skin wounds caused by *S. aureus* infection. Ruiz et al. [70] evaluated the phagocytic ability of human ASCs by flow cytometry, fluorescent latex beads, and transmission electron microscopy. The results showed that human ASCs had a strong phagocytic ability against common pathogenic microorganisms such as *S. aureus* in skin wounds. Wood et al. [71] also used scanning electron microscopy (SEM) and other techniques to evaluate human ASCs’ interaction with *S. aureus.* The results also showed that human ASCs had a strong phagocytosis effect on *S. aureus*. In addition, the growth and proliferation ability of *S. aureus* could be significantly inhibited even by using a conditioned medium obtained from ASCs without being infected with *S. aureus*, which was consistent with the results by Ruiz et al. The phagocytic ability of ASCs on pathogenic microorganisms such as *S. aureus* may be the intrinsic mechanism of ASCs in treating chronic skin wounds, providing a theoretical basis for future research on stem cell therapy in chronic skin wounds.

##### Bone and joint infection

Clinical treatment of bone and joint infection is limited. Currently, antibiotics and local debridement surgery are common strategies, but the treatment effects often fail to meet expectations. Therefore, cell therapy based on MSCs for bone infection has recently attracted wide attention. In the latest study in vitro, Yagi et al. [72] found that the conditioned medium of ASCs significantly inhibited the growth of *S. aureus* in joint synovial fluid. Previous studies have reported that the primary mechanism of antibacterial effect in MSCs is through cationic antimicrobial peptide LL-37 [73], which was further confirmed in this study. Pretreatment of ASC with 1, 25-dihydroxyvitamin D (1,25(OH)2D3) increased the expression of LL-37 and enhanced its antibacterial activity, and was reversed by vitamin D receptor antagonists [72], suggesting that the vitamin D signaling pathway plays a critical regulatory role.

Bone infection, which is most infected by *S. aureus* [74, 75], is a common cause of bone defects [76]. After infection, it mainly activates the NF-κB signaling pathway, reducing the proliferation of infected osteoblasts and their ability to form calcium nodules [77–79]. Meanwhile, activated B cells can secrete RANKL under infection, increasing the activity of osteoclasts [80]. All the above results in extensive bone loss after infection. To improve the prognosis of bone infection, stem cell therapy gradually comes into researchers’ vision in recent years. Wagner et al. [81] found that adequate debridement followed by topical application of ASCs in a mouse model of osteomyelitis resulted in increased osteoblast proliferation and reduced osteoclast numbers via the RANKL/OPG axis; In addition, the expression level of B-cell activating factor (BAFF) treated by ASCs in bone was significantly decreased, while the galectin-9 (GAL9) was increased. Immunofluorescence and flow cytometry showed that B cell populations decreased significantly. These results suggest that ASCs can regulate the innate immune system, improve the bone microenvironment and reduce the damage to bone regeneration after osteomyelitis. However, Seebach et al. [82] proposed the opposite view. Their study pointed out that BMSCs implantation on the femur of osteomyelitis rats significantly increased osteomyelitis score and aggravated infectious bone defects. In addition, they found that BMSCs exposed to *S. aureus* significantly increased the expression of pro-inflammatory factors such as IL-6, IL-1β, TNF-α, monocyte chemotactic protein-1 (MCP-1), and anti-inflammatory, immune mediators prostaglandin E synthase 3 (PTGES3) and TNF-stimulated gene 6 protein (TSG-6). However, the gene expression of the cathelicidin antimicrobial peptide (CAMP/LL-37), which was previously discussed as contributing to the cell-mediated bacterial defense mechanism, was not significantly enhanced. In conclusion, the application of MSC in infectious diseases still needs further research, but MSCs still have their unique therapeutic advantages in aseptic osteomyelitis. Meanwhile, MSCs conditional medium has been proven to have antibacterial activity, which could provide a new therapeutic strategy in bone infection.

##### Acute lung injury (ALI)

ALI is a clinical syndrome characterized by the acute onset of clinical symptoms such as tachypnea and hypoxemia. The mortality rate remained around 40% in the past 20 years [83, 84]. ALI can be caused by various causes, including *S. aureus* infection [85]. Qian et al. [86] studied the effect of ASCs on mice models of acute lung injury. They found that administrating ASCs through the airway significantly reduced the severity of lung inflammation and bacterial load of mice caused by *S. aureus* infection. It was also revealed that the antimicrobial activity of ASCs was mainly achieved through the secretion of TLR2-MyD88-JAK2/Stat3-dependent regenerating islet-derived IIIγ (RegIIIγ). Their findings revealed the therapeutic role of ASCs in acute lung injury caused by *S. aureus* infection and the internal mechanism of ASCs’ resistance to infection, indicating that ASCs played a bactericidal effect on *S. aureus* through the paracrine circuit. It suggests that ASCs may be a potential therapeutic target for *S. aureus*-caused ALI and provides a new strategy to treat ALI in the future.

##### Mastitis

*S. aureus*, the most common pathogen in bovine mastitis [87], can invade mammary epithelial cells, form abscesses and promote the formation of biofilms [88]. The effective rate of commonly used antibiotics (such as pirlimycin) in treating *S. aureus*-caused bovine mastitis is only 10-30% [89]. Therefore, sacrificing infected dairy cows is the most used strategy. But this strategy brings tremendous financial losses and seriously affects public health through unstable milk quality [90]. In the latest study, Peralta et al. [91] found that conditioned medium (CM) of MSC from fetal bovine bone marrow (BM-MSC) and adipose tissue (AT-MSC) significantly inhibited the growth of *S. aureus* isolated from clinical bovine mastitis cases in vitro. Among them, the primary AMP were β-defensin 4 A (bBD-4 A) and NK-lysine 1 (NK1). Still, only bBD-4 A was upregulated in BM-MSC, indicating differences in the antimicrobial efficacy of MSCs from different tissue sources against *S. aureus*. Torres et al. [92] verified the role of MSCs in treating *S. aureus*-caused mastitis in vivo. They found that the total bacterial load in milk from mastitis cows was significantly reduced after being treated with adipose tissue-derived stem cells (ASCs). Taken together, these results suggest a potential basis for the development of MSC-based therapies for mastitis. However, we should further explore the mechanism while optimizing its therapeutic effect.

#### Adipocyte progenitor cells (APCs)

In addition to ASCs, APCs are also one of the sources of mature adipocytes in biological adipose tissue [66]. The relationship between obesity and immune capacity has always attracted the attention of many researchers. Previous studies in vivo showed that the risk of bacterial infection in obese individuals was significantly higher than in normal individuals [93, 94]. By studying the infectious resistance in diet-induced obese mice infected with *S. aureus*, Zhang et al. [95] found that the proliferation and differentiation of APCs were abnormal in adipose tissue, which caused the depletion of APCs and accumulation of mature adipose cells during obesity. Moreover, ASCs in the adipose tissue in normal individuals can secrete AMP to resist *S. aureus* infection. Then they observed changes in AMP expression in adipocyte maturation and found that mature adipocytes could not produce AMP. They also demonstrated that mature adipocytes could indirectly inhibit ASCs through secreting transforming growth factor-β (TGFβ) by treating APCs with TGFβ-receptor inhibitors or peroxisome proliferator-activated receptor-γ agonists. These findings further suggest that the decreased resistance of obese individuals to *S. aureus* infection may be caused by the reduced ability of APCs to survive, multiply, and differentiate in adipose tissue. However, some studies found that the formation of dermal fat was conducive to enhancing the anti-infection ability of the body [96, 97], which needed further investigation.

With the continued understanding of the relationship between adipose-derived stem cells/ progenitor cells (ASPCs) and *S. aureus*, more and more studies have shown that ASPCs play an essential role in various inflammatory diseases caused by *S. aureus* (Fig. 2; Table 2). Although there are no clinical reports on using of ASPCs as the treatment of *S. aureus*-caused infectious diseases, the potential target role of ASPCs in the treatment of infectious diseases suggests that stem cell therapy may be a new therapeutic approach for delayed wound healing and soft tissue inflammation caused by *S. aureus* infection in the future.

**Table 2:** The role of mesenchymal, adipose-derived, dental pulp, umbilical cord stem cells and adipose-derived progenitor cells on diseases associated with *S. aureus*-related infection

Cell type	Functional components	Type of Disease	Outcomes
Mesenchymal stem cells (MSCs)	Antimicrobial peptides(AMPs)LL37 and cytokines produced by hMSCs	Cystic fibrosis(CF)	Protection against CF lung infection caused by *S. aureus* [51]
	AMPs, cysteine protease, CCL2 and cytokines produced by hMSCs	Chronic skin injury	Inhibition of local colonization of *S. aureus* and promote wound healing [61, 62]
	Unknown	Bone and joint infections	Increased the severity of *S. aureus* infection and infective bone defect [82]
	β-defensin 4 A (bBD-4 A) produced by BM-MSCs	Mastitis	Inhibition of the activity of *S. aureus* [91]
Adipose-derived stem cells(ACSs)	Unknown	Chronic skin injury	Promoted healing of wounds infected with type 2 diabetes [69]
	LL37 and other AMPs	Bone and joint infections	Inhibition of the growth of *S. aureus* in joint synovial fluid [72]
	Unknown		Protection of the bone microenvironment and promote bone tissue regeneration [81]
	Regenerating islet-derived IIIγ (RegIIIγ)	Acute lung injury(ALI)	Reduced the severity of lung inflammation and bacterial load caused by *S. aureus* infection in mice [86]
	bBD-4 A and NK-lysine 1 (NK1)	Mastitis	Reduced the severity and bacterial load of mastitis in cattle caused by *S. aureus* [91, 92]
Adipose-derived progenitor cells(APCs)	AMPs	Diseases associated with Staphylococcus aureus infection	Protection against *S. aureus*-related infection [95]
Umbilical cord stem cells	Unknown	Severe pneumonia	Alleviated the clinical symptoms and lung lesions of severe pneumonia in rabbits [107]
Dental pulp stem cells	Hepatocyte growth factor(HGF)	Oral infectious diseases	Inhibition of the activity of *S. aureus* and promoted pulp repair and regeneration [116]

LL37: Antibacterial Protein LL-37; CCL2: Chemokine (C-C motif) ligand 2; bBD-4 A: β-defensin 4 A;

### Other stem cells

#### Human umbilical cord-derived mesenchymal stem cells (hucMSCs)

In addition to ASPCs, umbilical cord-derived stem cells, also known as hucMSCs, are a particular type of MSCs. hucMSCs can be isolated from the human umbilical cord with low immunogenicity, which makes hucMSCs a promising candidate for stem cell therapy [98].

MRSA is one of the main pathogenic microorganisms of hospital infection [99], and severe pneumonia caused by MRSA often induced cytokine storm, eventually leading to multiple organ dysfunction syndromes (MODS) and even death [1, 100]. Linezolid is the preferred antibiotic in hospital-acquired pneumonia [101, 102]. Still, due to the imbalance between the pathogen and the host immune system during pneumonia, the therapeutic effect of antibiotics alone could be better [103]. MSCs have become one of the essential therapeutic methods in regenerative medicine in recent years due to their powerful immunomodulatory properties and their ability to ameliorate the storm of inflammatory factors through paracrine [104–106]. KONG et al. [107] found that in a rabbit model of severe pneumonia, the combination treatment of hucMSCs and linezolid significantly alleviated clinical symptoms such as cough, shortness of breath, decreased food intake, and less mucosal congestion and erosions under bronchoscopy. They also found that immune cell infiltration and inflammatory exudation were more limited, and the plasma levels of IL-8, IL-6, C-reactive protein (CRP), and TNF-α significantly decreased. Based on these findings, Mccarthy et al. [108] proposed that MSCs could be delivered directly to the lungs of infected people by atomized cell suspension. After atomization, the antibacterial capacity and the contents of factors such as hepcidin, lipid carrier protein-2, and IL-8 were not affected, but it was worth noting that the LL-37 level was significantly reduced. This may be related to the gas-liquid interface generated by the atomizer, which may affect protein stability [109]. In conclusion, hucMSCs and linezolid administration improve the survival rate of severe pneumonia and reduce lung impairment (Fig. 2; Table 2). In addition, Nebulization technologies provide us with new clinical insights. These findings highly indicated the clinical potential of MSCs in treating severe pneumonia.

#### DPSCs

By studying human DPSCs, Gronthos et al. [110] found a kind of cells that have similar immunophenotype with BMSCs and can form mineralized nodules. The cells, with spindle morphology, self-renewal ability, and multiple differentiation potential, isolated from pulp tissue, are called DPSCs. During infection and injury, DPSCs enhance their proliferation and differentiation ability and stimulate the migration of pulp progenitor cells to the site, where they generate a protective layer to protect and repair dentin [111–113]. Therefore, their potential therapeutic role in dental caries and other oral diseases has attracted increasing attention from researchers.

The reaction of dental pulp to dental caries is a complex process to prevent dental caries’ lesions and protect the dental pulp from bacterial invasion [114]. DPSCs differentiate into new odontogenic cells to accomplish repair, regeneration, and tertiary dentin formation. Since the elimination of local infection occurs before repair and regeneration, most people believe that DPSCs have both abilities to regenerate and antibacterial potential [115]. Lundy et al. [116] confirmed that, unlike previous studies, the classical AMP (LL-37, β-defensin 2, β-defensin 3, and lipocalin) had lower gene expression in DPSCs. However, they found that hepatocyte growth factor (HGF) in the culture medium of DPSCs can destroy the bacterial membrane of *S. aureus in vitro*, thereby exerting its bactericidal activity (Fig. 2; Table 2). In conclusion, with the deepening of our understanding of the antibacterial ability of DPSCs, more support will be provided for dental pulp repair and regeneration in the future.

## Conclusions and future perspectives

This paper reviewed the relationship between *S. aureus* and stem cells. We found that *S. aureus* increased the migration, proliferation, and osteogenic differentiation of MSCs and increased the proliferation and differentiation of other stem cells, such as HSPCs and DPSCs, in the case of acute infection. However, when the infection progressed to the chronic stage, *S. aureus* could inhibit the average physiological ability of stem cells such as MSCs, HSPCs, and DPSCs, even leading to permanent destruction of stem cells (such as FD). It showed that the effect of *S. aureus* on stem cells was closely related to the toxicity and infection severity of bacteria strain. In addition, we also found that bone marrow MSCs, ASPCs, DPSCs, umbilical cord stem cells, and other stem cells had specific therapeutic effects on various *S. aureus*-caused infection. These stem cells could directly or indirectly target *S. aureus* and biofilm and then killed *S. aureus* and relived the local inflammation.

However, stem cells still face many challenges in the treatment of infectious diseases associated with *S. aureus*: (1) Complexity of Mechanisms: The intricacies of stem cell therapy in combating infections surpass those of conventional antibiotic therapy. Stem cells exhibit a diverse range of mechanisms including direct antimicrobial activity, immune response modulation, and tissue repair following infection-induced damage [117, 118]. The multifaceted nature of these mechanisms presents obstacles for both research and practical implementation in clinical settings. (2) Evaluation of efficacy: The evaluation of the efficacy of stem cell therapy is difficult. For *S. aureus*-related infections, traditional assessment metrics focus on pathogen clearance and reduction of infection markers. However, stem cell therapy may affect multiple biological processes simultaneously, including immunomodulation and tissue repair, so more comprehensive methods of efficacy assessment need to be developed [92, 119]. (3) Safety concerns: The safety implications of utilizing stem cell therapy in the context of infectious diseases are of particular concern. For instance, while stem cells may facilitate tissue regeneration, they may also hinder the immune system’s ability to combat pathogens, potentially resulting in the prolonged presence or reemergence of the infection [120, 121]. (4) Antibiotic resistance: Staphylococcus aureus, particularly methicillin-resistant strains, have demonstrated significant resistance to various antibiotics [122]. The potential incorporation of stem cell therapy in addressing this issue may necessitate a concurrent use of antibiotics; however, the optimal strategy for this combination to prevent the emergence of resistance remains a subject of inquiry [123–125]. (5) Individual differences: Variability among patients, such as differences in immune status, pathogen characteristics, and the location and severity of infection, can impact the effectiveness of stem cell therapy [126, 127]. Consequently, personalized treatment regimens are necessary, leading to heightened complexity in clinical implementation. (6) Ethical and regulatory: The ethical and regulatory issues associated with stem cell therapy apply equally to the use of stem cells in the treatment of *S. aureus*-related infections. In particular, research using embryonic stem cells may face more stringent ethical scrutiny and legal restrictions.

In summary, *S. aureus* and its related components affect the functional state of various stem cells, while the stem cells secrete polypeptides, chemokines and cytokines (AMP, HGF, etc.) that inhibit the activity of *S. aureus*. Therefore, further studies on the interaction between *S. aureus* and stem cells may provide new ideas for the treatment of infectious diseases in the future, but their clinical efficacy and safety need to be further investigated and confirmed.

## Data Availability

Not applicable.

## Abbreviations

S. aureus: Staphylococcus aureus
MRSA: Methicillin-resistant S. aureus
iPSCs: Induced pluripotent stem cells
ESCs: Embryonic stem cells
MSCs: Mesenchymal stem cells
BMSCs: Bone marrow-derived mesenchymal stem cells
TNF-α: Tumor necrosis factor α
CCL20: C-C motif chemokine ligand 20
SDF-1: Stromal derived factor 1
VEGF: Vascular endothelial growth factor
adMSCs: Adipose-tissue-derived mesenchymal stem cells
ALP: Alkaline phosphatase
LTA: Lipoteichoic acid
SEC2: Staphylococcal enterotoxin C2
Runx2: Runt-related transcription factor-2
ColI: Collagen type I
HSPCs: Hematopoietic stem cells and progenitor cells
HCSs: Hematopoietic stem cells
HPCs: Hematopoietic progenitor cell
HMSCs: Human mesenchymal stem cells
ASCs: Adipose tissue-derived stem cells
E.V.s: Extracellular vesicles
IL-1: Interleukin-1
TLR2: Toll-like receptor 2
MyD88: Myeloid differentiation factor 88
PGE2: Prostaglandin E2
DPSCs: Dental pulp stem cells
CF: Cystic fibrosis
CFTR: Cystic fibrosis transmembrane conduction regulator
HDL: High-density lipoaspirate
SEM: Scanning electron microscopy
BAFF: B-cell activating factor
GAL9: Galectin-9
MCP-1: Monocyte chemotactic protein-1
TSG-6: TNF-stimulated gene 6 protein
PTGES3: Prostaglandin E synthase 3
RegIIIγ: Regenerating islet-derived IIIγ
bBD-4A: β-defensin 4 A
NK1: NK-lysine 1
APCs: Adipocyte progenitor cells
TGFβ: Transforming growth factor-β
ASPCs: Adipose-derived stem cells/ progenitor cells
HucMSCs: Human umbilical cord-derived mesenchymal stem cells
CRP: C-reactive protein
